# Supplementary material for: Content-rich biological network constructed by mining PubMed abstracts
Source: BMC Bioinformatics. 2004 Oct 8;5:147. doi: 10.1186/1471-2105-5-147 (PMC528731; doi:10.1186/1471-2105-5-147)
Supplement: Additional File 2 — The original results of the above study (non-essential files are deleted to keep the file size under the limit set by BMC bioinformatics). [file 1471-2105-5-147-S2.bz2 › chilibotAdditionalFile2/dip05/750ID9353297E18133/html/FHUA_TONB.html]

 


 **FHUA** and **TONB** 
  
Found 56 abstracts in PubMed, retrieved 05.  
 

 What does Google say? 
 PDF only 
| .edu only 

---

**Interactive relationship** (e.g. stimulation, inhibition, etc)

**Neutral relationship**- **TonB**  of Escherichia coli activates  **FhuA**  through interaction with the beta barrel.  Ref: 12427941 Microbiology, 2002
- these differences might contribute to the lower activity of the P. agglomerans  **TonB**  protein when coupled to the E. coli  **FhuA**  protein.  Ref: 12427941 Microbiology, 2002
- The data support the theory that the beta barrel receives the energy from the cytoplasmic membrane via  **TonB**  and responds to the energy input and thus represents the transporting domain of  **FhuA** .  Ref: 12427941 Microbiology, 2002
- However, deletion of the  **TonB**  box along with the globular domain results in a protein, FhuAdelta5 160, that still displays  **TonB**  dependent active ferrichrome transport across the outer membrane and confers sensitivity to the  **FhuA**  ligands.  Ref: 12427941 Microbiology, 2002
- The energy source for active transport is the proton motive force of the cytoplasmic membrane, which is required for all  **FhuA**  functions except infection by phage T5, and is thought to be mediated to the outer membrane receptor  **FhuA**  by the  **TonB**  protein.  Ref: 12427941 Microbiology, 2002
- **TonB**  missense mutants reduced the activity of  **FhuA**  and FhuAdelta5 160.  Ref: 12427941 Microbiology, 2002

**Non-interactive relationship** (e.g. studied together, co-existance, homology, etc.)

- **TonB**  proteins of different Enterobacteriaceae activated  **FhuA**  and FhuAdelta5 160 to a similar degree.  Ref: 12427941 Microbiology, 2002
- The crystallographic structures of two  **TonB**  dependent receptors  **FhuA**  and FepA have recently been determined.  Ref: 12409204 Biochim Biophys Acta, 2002
- We consider a variety of different colicins, including A, B, D, E1, E3, Ia, M and N, that penetrate through the porins OmpF, FepA, BtuB, Cir and  **FhuA** , to subsequently interact with a few targets in the periplasm, including TolA, TolB, TolC and  **TonB** .  Ref: 12423783 Biochimie< MedlineTA>Biochimie, 2002
- In this study synthetic nonapeptides identical in sequence to amino acids 150 15 51 15 52 16 53 161 and 158 166 of  **TonB**  were shown to reduce ferrichrome transport of cells via wild type  **FhuA**  and the corkless derivative FhuAdelta5 160, which suggests that this  **TonB**  region is involved in the interaction of  **TonB**  with the beta barrel of  **FhuA** .  Ref: 12427941 Microbiology, 2002
- DALTV of FecA could be functionally replaced with the core  **TonB**  boxes of  **FhuA**  DTITV and FepA DTIVV.  Ref: 12618451 J Bacteriol, 2003
